# Regulation of Avian Leukosis Virus Subgroup J Replication by Wnt/β-Catenin Signaling Pathway

**DOI:** 10.3390/v13101968

**Published:** 2021-09-30

**Authors:** Dandan Qiao, Qian He, Xiaowei Cheng, Yongxiu Yao, Venugopal Nair, Hongxia Shao, Aijian Qin, Kun Qian

**Affiliations:** 1 Ministry of Education Key Lab for Avian Preventive Medicine, Yangzhou University, No. 48 East Wenhui Road, Yangzhou 225009, China; qiaodandan20210603@163.com (D.Q.); yzuheqian@163.com (Q.H.); huaqiangxuwei@126.com (X.C.); hxshao@yzu.edu.cn (H.S.); aijian@yzu.edu.cn (A.Q.); 2 School of Animal Engineering, Xuzhou Vocational College of Bioengineering, Xuzhou 221006, China; 3 Jiangsu Key Lab of Preventive Veterinary Medicine, Yangzhou University, No. 48 East Wenhui Road, Yangzhou 225009, China; 4 The Pirbright Institute & UK-China Centre of Excellence for Research on Avian Diseases, Pirbright, Surrey GU24 0NF, UK; yongxiu.yao@pirbright.ac.uk (Y.Y.); venugopal.nair@pirbright.ac.uk (V.N.); 5 The International Joint Laboratory for Cooperation in Agriculture and Agricultural Product Safety, Ministry of Education, Yangzhou University, Yangzhou 225009, China

**Keywords:** avian leukosis virus subgroup J, Wnt/β-catenin signal pathway, GSK-3 inhibitor, iCRT14, virus replication

## Abstract

Wnt/β-catenin signaling is a highly conserved pathway related to a variety of biological processes in different cells. The regulation of replication of various viruses by Wnt/β-catenin signaling pathway has been reported. However, the interaction between the Wnt/β-catenin pathway and avian leukosis virus is unknown. In the present study, we investigated the effect of modulating the Wnt/β-catenin pathway during avian leukosis virus subgroup J (ALV-J) infection. The activation of the Wnt/β-catenin pathway by GSK-3 inhibitor increased ALV-J mRNA, viral protein expression, and virus production in CEF cells. This increase was suppressed by iCRT14, one of the specific inhibitors of the Wnt/β-catenin signaling pathway. Moreover, treatment with iCRT14 reduced virus titer and viral gene expression significantly in CEF and LMH cells in a dose-dependent manner. Inhibition Wnt/β-catenin signaling pathway by knockdown of β-catenin reduced virus proliferation in CEF cells also. Collectively, these results suggested that the status of Wnt/β-catenin signaling pathway modulated ALV-J replication. These studies extend our understanding of the role of Wnt/β-catenin signaling pathway in ALV-J replication and make a new contribution to understanding the virus–host interactions of avian leukosis virus.

## 1. Introduction

Avian leukosis virus (ALV) belongs to the genus *Alpharetrovirus* of the family *Retroviridae*, which causes immunosuppression, reproductive disorders, and a diverse set of tumors, causing significant economic losses in the poultry industry worldwide [1,2,3]. Within the five subgroups of exogenous ALVs, subgroup J (ALV-J) is the major ALV affecting poultry health in China. It can induce a spectrum of different neoplasms in meat-type and layer chickens. In addition, ALV-J infection is prevalent in the Chinese native breeds of chickens [3,4]. To date, there are no effective preventive vaccines to protect chicken against ALV-J infection. In recent years, several groups reported the significance of the host’s innate immune responses during ALV-J infection. These reports demonstrated that interferon-stimulated genes (ISGs) were not induced in birds with hemangioma due to the reduction of IRF1 and STAT1 gene expression [5]. Similarly, investigation into the interaction between ALV-J and macrophages by transcriptome analysis [6] showed that ALV-J infection induced the upregulation and blocking of the phosphorylation of IkB [7]. However, there are still many unknowns regarding the virus–host interactions of ALV-J.

The canonical Wnt/β-catenin signaling pathway plays an important role in cell development and differentiation. It is closely associated with developmental diseases and cancer [8,9,10]. In the absence of the Wnt signaling pathway ligand in cells, β-catenin binds to the axonal protein (AXIN)-mediated cytoplasmic complex, is phosphorylated by glycogen synthase kinase-3β (GSK3β), and then is ubiquitinated and degraded by proteasome, resulting in low levels of β-catenin in the cytoplasm. When the Wnt signal is in the “on” state, the AXIN/GSK3β mediated complex is dissociated, resulting in the stabilization and accumulation of β-catenin in the cytoplasm and translocation into the cell nucleus. Subsequently, β-catenin interacts with the T-cell factor/lymphoid enhancer binding factor 1 (TCF/LEF) and initiates the downstream transcription of genes such as c-myc, cyclinD1, and matrix metalloproteinases (MMPs) [11,12,13].

Several reports have demonstrated the interaction of the Wnt/β-catenin signaling pathway with human and animal viruses, including human immunodeficiency virus (HIV) [14], herpes simplex virus 1 (HSV1) [15], hepatitis B virus (HBV) [16], hepatitis C virus (HCV) [17], influenza virus (IAV) [18], porcine reproductive and respiratory syndrome virus (PRRSV) [19,20], porcine circovirus-like virus P1 [21], bovine parainfluenza virus type 3 [22], and bovine herpesvirus 1 [23]. Some of these viruses activate the Wnt/β-catenin signaling pathway, while others deactivate it through diverse mechanisms. However, its role in avian virus infection remains largely unknown, although some studies showed that Wnt/β-catenin pathway activation was required for chicken primordial germ cells proliferation [24] and growth retardation induced by ALV-J infection [25].

In this study, we investigated for the first time the relationship between the Wnt/β-catenin signaling pathway and ALV-J replication in CEF cells. Our findings provide new insights into the virus–host interaction of avian leukosis virus.

## 2. Materials and Methods

### 2.1. Virus, Cells, and Reagents

The JS09GY3 (GenBank ID: GU982308) strain of ALV-J was maintained at the Ministry of Education Key Lab for Avian Preventive Medicine, Yangzhou University. Chicken embryo fibroblast (CEF) cells were isolated from 9-day-old SPF chicken embryos. The cells were grown in Dulbecco’s modified Eagle medium (DMEM; GIBCO, Shanghai China) supplemented with 5% fetal bovine serum (FBS), 100 U/mL penicillin, and 100 g/mL streptomycin at 37 °C in a 5% CO_2_ atmosphere. LMH (ATCC^®^ cRL2117™), a primary hepatocellular carcinoma epithelial cell line of a leghorn chicken, was maintained in F12 medium (GIBCO, Shanghai China) supplemented with 10% fetal bovine serum (FBS), 100 U/mL penicillin, and 100 g/mL streptomycin at 37 °C in a 5% CO_2_ atmosphere. GSK-3 inhibitor X from Merck (Shanghai, China) was diluted in dimethyl sulfoxide (DMSO). iCRT14 was purchased from SIGMA (Shanghai, China) and diluted in dimethyl sulfoxide (DMSO). Lipofectamine RNAiMax and 2000 reagent were purchased from Invitrogen (Shanghai, China). The Cell Counting Kit-8 (CCK-8) for quantitation of viable cell number in proliferation and cytotoxicity assays was purchased from Vazyme Biotech company (Nanjing, China). The specific monoclonal antibodies, 5D3 and JE9, against ALV-J p27 and gp85 protein, respectively, were described in our previous reports [26]. Affinity-purified rabbit polyclonal antibody 5527 against chicken β-catenin was generated by Gene Script Bioscience and Technology Company (NanJing, China) by immunization with peptides PGDSNQLAWFDTDLC). The anti-α-tubulin antibody was purchased from SIGMA (Shanghai, China). TCF Reporter Plasmid Kit was purchased from Merck (Shanghai, China).

### 2.2. Quantitative Reverse-Transcriptase Polymerase Chain Reaction

The relative expression levels of the gp85, p27, TCF4, LEF1, Axin, APC, β-catenin, cyclin D1, c-myc, and 18S were determined by real-time PCR (7500 Real-Time PCR System, ABI, Shanghai, China) as reported previously [27]. The sequences of the primers listed in Table 1 were synthesized by Gene Script Bioscience and Technology Company (NanJing, China).

The total RNA from cells was prepared by using the AxyPrep™ Multisource Total RNA Miniprep kit (AXYGEN, Hangzhou, China), and 1 μg RNA was used in reverse-transcription reaction using PrimeScript RT Master Mix (TaKaRa, Dalian, China) following the manufacturer’s instructions. Here, 1 μL diluted cDNA, 400 nM primers, and 10 μL SYBR Green Master Mix were used for the real-time PCR in a final volume of 20 μL. The amplification conditions were as follows: 95 °C for 30 s, followed by 40 cycles of 95 °C for 5 s and 60 °C for 34 s. Dissociation curves were generated to analyze the individual PCR products. The gene expression levels were normalized to the level of chicken 18S mRNA. The analyses of the relative gene expression data were performed by using the 2^−^^ΔΔCT^ method. The results are presented as fold-change of relative expression compared with the control group.

### 2.3. Dual-Luciferase Reporter Assay

To evaluate β-catenin/TCF transcriptional activity, CEF cells were transfected with the luciferase reporter plasmids pTOP FLASH, which contains wild-type TCF binding sites (Merck, Shanghai, China) and pFOP FLASH, which contains mutant TCF binding sites (Merck, Shanghai, China), as described previously [17,20,28]. CEF cells transfected with Top flash or Fop flash were inoculated with GSK3β inhibitor or iCRT14 to test whether TCF/LEF activation could be regulated in CEF cells. Cells in 48-well plates were transfected using lipofectamine 2000 reagent (Invitrogen, Sahnghai, China) with either 0.2 μg Top flash or Fop flash together with 0.02 μg pRL-TK. At 24 h post-transfection, the cells were treated by 5 μM GSK-3 inhibitor with or without 10 μM iCRT14. Cell extractions were collected at 24 h after treatment. Luciferase activity was monitored using a dual-luciferase reporter assay system (Promega, Sahnghai, China) following the manufacturer’s protocol. The values were normalized with respect to Renilla luciferase activity.

### 2.4. Cell Proliferation and Cytotoxicity Assays

The proliferation and cytotoxicity of iCRT14 on CEF and LMH cells were determined using a cell counting kit, CCK-8 kit (Vazyme, Nanjing, China). In brief, 1.0 × 10^4^/well CEF cells were seeded in 96-well plates, and 100 μL of iCRT14 at 0 and 10 μM in DMEM maintenance medium were added to each well. After incubating for 24, 48, 72, and 96 h in a 37 °C incubator, CCK-8 solution (10 μL) was added to each well. Incubation was continued for 1 h, and the absorbance at 450 nm was measured.

### 2.5. Western Blot Analysis

As previously reported [26], the cells were lysed with RIPA buffer, and the protein concentrations were determined with a BCA protein assay kit (Bio-Rad, Sahnghai, China). The proteins (30 μg) were denatured by heating (5 min, 100 °C) and were separated in 12% SDS-PAGE under reducing conditions. The proteins in the gels were electrotransferred to nitrocellulose membranes (Sigma, Shanghai, China). The membranes were blocked with 5% skim milk in PBST (PBS containing 0.1% Tween 20) for 1 h at room temperature and probed for 2 h at room temperature with the appropriate primary antibodies. The blots were washed three times with PBST and incubated with the appropriate HRP-conjugated secondary antibody (SIGMA, Shanghai China) for 60 min at room temperature. The blots were then washed three more times and developed using an enhanced chemiluminescence (ECL) detecting system (Pierce, Rockford, IL, USA).

### 2.6. Inhibition of β-Catenin

Previously, we demonstrated that the small interfering RNA (siRNA) 986 (sense 5′-GGACCUACACUUAUGAGAATT-3′, antisense 5′-UUCUCAUAAGUGUAGGUCCTT-3′) designed by Genepharma company (Shanghai, China) showed interference capacity above 50% (unpublished data). In the present study, the CEF cells were seeded in 12-well plates (4 × 10^5^cells/well). Cells were transfected with RNA oligos siRNA 986 or a control siRNA at 140 pmol using Lipofectamine RNAiMax. Then, 24 h after transfection, CEF were infected with ALV-J at MOI of 1.0 and cultured for 72 h. Cell lysates were then collected for Western blot assay, and RNA were extracted for qRT-PCR. The virus titer in the culture supernatant was determined by TCID_50_ assay.

### 2.7. Confocal Microscopy

This was performed as described in our previous study [26]. In brief, cells on coverslips were fixed with 4% paraformaldehyde in phosphate-buffered saline (PBS) for 20 min at room temperature, permeabilized with 0.25% Triton X-100 for 5 min, washed with PBS, blocked with 2% BSA for 30 min, and incubated with anti-p27 mAb 5D3 (5 μg/mL) or antichicken β-catenin pAb 5527 (5 μg/mL) in PBS for 45 min at room temperature. The cells were then washed in PBS and incubated with FITC conjugated goat antimouse or goat antirabbit IgG (Sigma, Sahnghai, China) and stained with 10 μg/mL Hoechst 33,342 dye (SIGMA, Shanghai, China) at room temperature for an additional 30 min. The pictures were captured with a Leica SP2 confocal microscope.

### 2.8. Virus Titration

The TCID_50_ assay was determined to evaluate virus titer. CEF cells were cultured in 96-well plates at a density of 4 × 10^4^ cells per well. In addition, 10-fold serially diluted virus samples were added into each well at 100 μL in eight replicates. The cells were then incubated for 5 days, and the TCID_50_ was calculated using the Reed–Muench method. Each virus titration assay was performed in triplicate.

### 2.9. Statistical Analyses

The data were expressed as means ± standard deviations (SD) of triplicate determinations. The significance of the variability between the trials was analyzed using the GraphPad prism (version 5.0) software. Differences in data were evaluated by the Student’s *t* test. Significant differences were considered at *p* < 0.05.

## 3. Results

### 3.1. Activation of Wnt/β-Catenin Signaling Pathway Enhances ALV-J Replication and Proliferation

GSK-3 inhibitor X, an acetoxime analog of 6-bromoindirubin-3′-oxime (BIO), has been utilized to activate Wnt/β-catenin signaling by stabilizing β-catenin from the degradation complex in mammalian studies [28,29]. However, there is no such reference study in chicken cells. We first determined the effects of GSK-3 inhibitor X on the Wnt/β-catenin signaling pathway in CEF cells.

**Figure 1:**
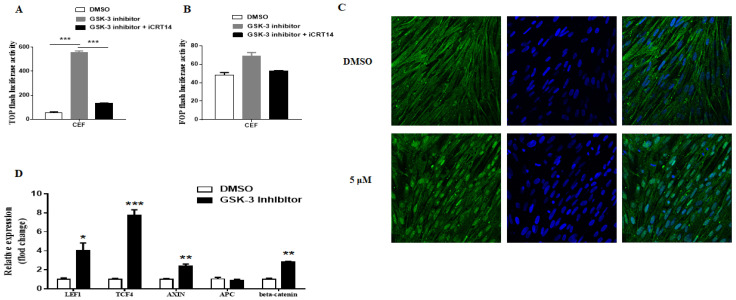
GSK-3 inhibitor activated the Wnt/β-catenin signaling pathway in CEF cells. Relative luciferase activity of pTOP (**A**) and pFOP (**B**) in CEF cells. GSK-3 inhibitor enhanced the β-catenin/Tcf-dependent transcriptional activity in CEF cells. (**C**) After treatment with GSK-3 inhibitor for 24 h, the cells were fixed and stained for β-catenin (green) and the nuclei (blue). The pictures were captured and merged with a Leica SP2 confocal microscope (magnification, ×630). (**D**) The gene expression levels of Wnt/β-catenin signaling-pathway-associated genes were quantified by real-time RT-PCR. Data were expressed as mean ± SD from three independent experiments and analyzed by Student’s *t*-tests (* *p* < 0.05, ** *p* < 0.01, *** *p* < 0.001). These experiments were performed independently at least three times with similar results.

By using the Wnt/β-catenin signaling TOP/FOP flash reporter assay, we first validated that GSK-3 inhibitor X could activate the Wnt/β-catenin signaling in CEF cells. In addition, the activation could be restored by iCRT14, one of the specific small molecular inhibitors of β-catenin responsive transcription (Figure 1A,B). After 24 h of GSK-3 inhibitor X (5 μM) treatment, the β-catenin translocated to the nucleus compared with the DMSO control group by confocal microscopy (Figure 1C). The RNAs were extracted for real-time PCR to detect Wnt/β-catenin signaling-associated genes expression. The result clearly showed that TCF4, LEF, Axin, and β-catenin gene expression level were significantly enhanced after GSK-3 inhibitor X treatment (Figure 1D). Overall, these results indicated that GSK-3 inhibitor X could activate the Wnt/β-catenin signaling pathway in CEF cells.

**Figure 2:**
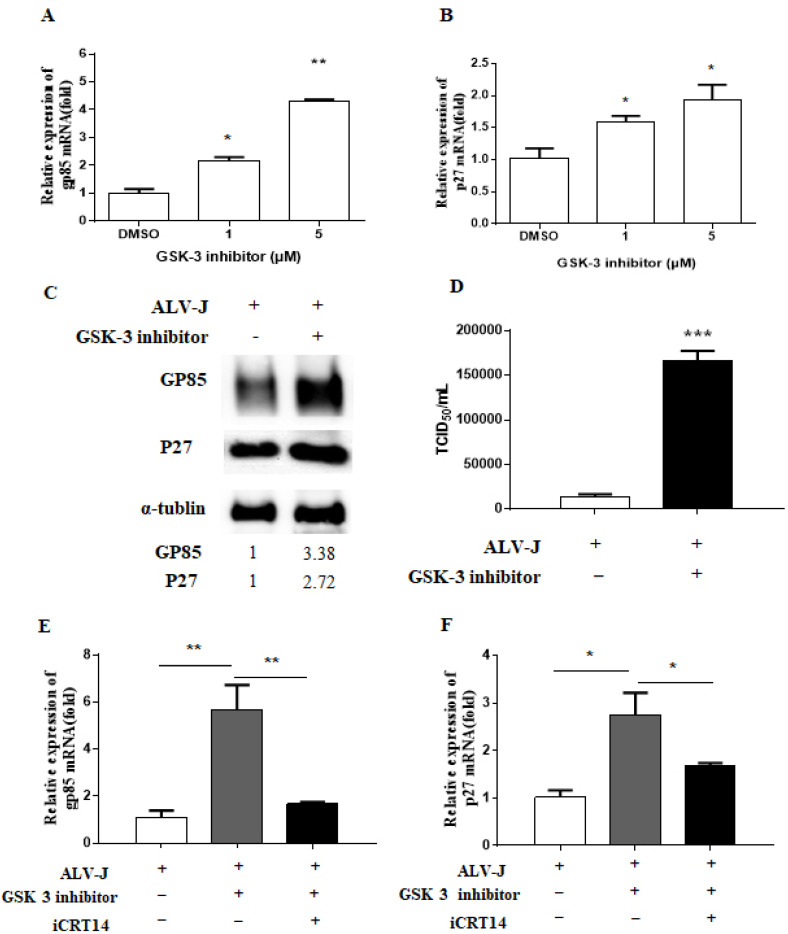
GSK-3 inhibitor induced ALV-J replication via the Wnt/β-catenin signaling pathway. Effects of GSK-3 inhibitor on the expression levels of the gp85 (**A**) and p27 (**B**) genes were quantified by real-time RT-PCR. (**C**) The viral gp85 and p27 protein levels were measured by immunoblotting at 72 h postinfection. The control α-tubulin levels indicate that similar levels of protein were loaded into each lane, and the grayscale value of the bands in Western blot were analyzed with Image J software. (**D**) Virus production was analyzed by virus titration assay in CEF cells treated with 5 μM GSK-3 inhibitor and DMSO control. The gene expression levels of gp85 (**E**) and p27 (**F**) induced by GSK-3 inhibitor were restored by iCRT14. Data were expressed as mean ± SD from three independent experiments and analyzed by Student’s *t*-tests (* *p* < 0.05, ** *p* < 0.01, *** *p* < 0.001). These experiments were performed independently at least three times with similar results.

CEF cells were pre-treated with GSK-3 inhibitor X (5 μM) for 3 h and then infected with ALV-J JS09GY3 at MOI of 1.0. After 72 h of inoculation, the RNA and cell lysates were collected for real-time PCR and Western blotting assay, respectively. We found that the transcription of the gp85 and p27 genes were significantly increased in a dose-dependent manner, compared with the control DMSO group (Figure 2A,B). The treatment with GSK-3 inhibitor X also increased gp85 and p27 protein expression as determined by Western blotting assay (Figure 2C). In addition, the virus titer in culture medium was increased also using TCID_50_ assay (Figure 2D). Moreover, the upregulated viral gene expression levels were recovered by iCRT14, one of the specific inhibitors of the Wnt/β-catenin signaling pathway (Figure 2E,F). These results indicated that activation of Wnt/β-catenin signaling significantly increased ALV-J replication in CEF cells.

### 3.2. Inhibition of Wnt/β-Catenin Signaling Pathway Reduces ALV-J Replication and Proliferation

**Figure 3:**
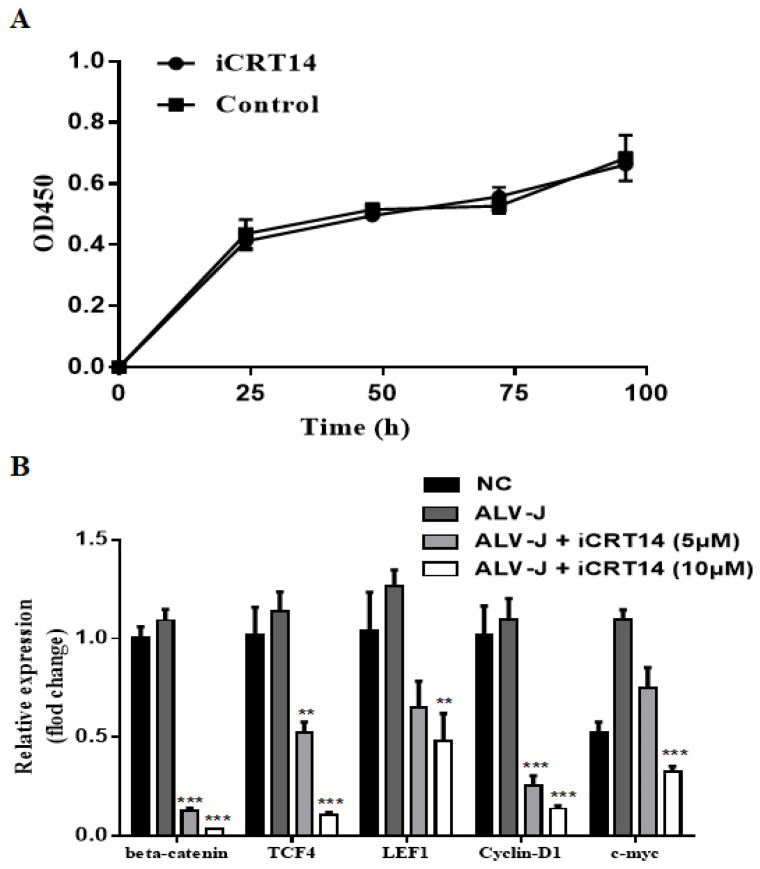
iCRT14 inhibited the Wnt/β-catenin signaling pathway in CEF cells. (**A**) CEF cells were treated with iCRT14 (10 μM) or vehicle control for various times (24, 48, 72, 96 h), and the cell viability was detected by OD450 with CCK-8 kit. Data are expressed as the mean ± SD of three independent experiments. (**B**) The gene expression levels of Wnt/β-catenin signaling-pathway-associated genes and target genes were quantified by real-time RT-PCR. Data were expressed as mean ± SD from three independent experiments and analyzed by Student’s *t*-tests (** *p* < 0.01, *** *p* < 0.001). These experiments were performed independently at least three times with similar results.

According to the results shown in Figure 1A, we chose iCRT14, a Wnt signaling inhibitor that disrupts the interaction between β-catenin and TCF4 [30], to examine the effect on ALV-J replication in CEF. We first validated that iCRT14 inhibited the Wnt/β-catenin signaling pathway by crippling pathway-associated gene expression at a noncytotoxic concentration (5 and 10 μM) in CEF cells (Figure 3A,B).

**Figure 4:**
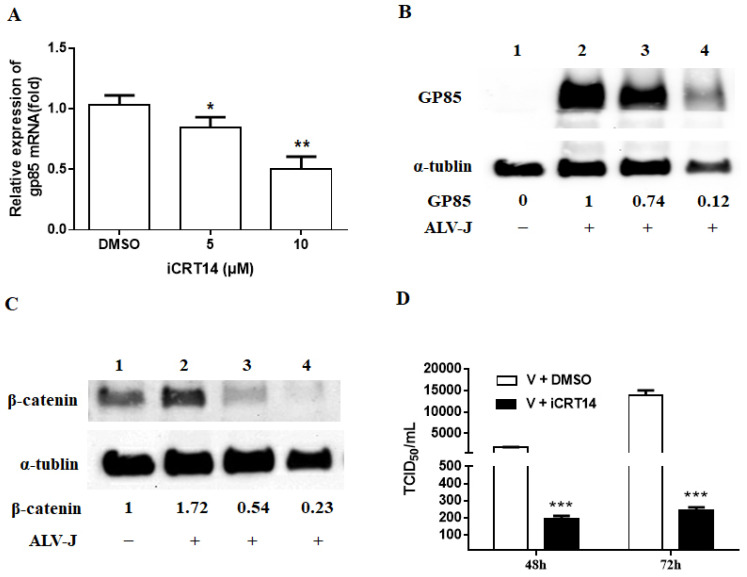
iCRT14 decreased ALV-J replication in CEF cells. (**A**) After 72 h of incubation, effects of iCRT14 on the expression levels of the gp85 gene were quantified by real-time RT-PCR. In addition, the DMSO group served as the control. Differences in gene expression between the groups were assessed with an analysis of variance followed by Student’s t-tests. Data were expressed as mean ± SD from three independent experiments and analyzed (* *p* < 0.05, ** *p* < 0.01). The protein levels of viral encoded gp85 (**B**) and β-catenin in cell (**C**) were measured by immunoblotting at 72 h p.i. with iCRT14 treatment. The control α-tubulin levels indicate that similar levels of protein were loaded into each lane, and the grayscale value of the bands in Western blot were analyzed with Image J software. Line 1: Nonvirus control group; line 2: DMSO group; line 3: 5 μM iCRT14 group; and line 4: 10 μM iCRT14 group. The immunoblotting experiments were performed independently at least three times with similar results. (**D**) Virus proliferation was analyzed by TCID_50_ assay in CEF cells treated with 10 μM iCRT14 and DMSO control. Data were expressed as mean ± SD from three independent experiments and analyzed by Student’s *t*-tests (*** *p* < 0.001). These experiments were performed independently at least three times with similar results.

CEF cells were then treated with gradient concentration (5 and 10 μM) for 4 h before being infected with ALV-J JS09GY3 at MOI of 1.0. After a 72 h incubation, the RNA and cell lysates were collected for real-time PCR and Western blotting assay, respectively. The results showed that the transcription of the gp85 gene were significantly reduced in a dose-dependent manner compared with the DMSO control group (Figure 4A). A similar result was also observed with the protein level by Western blotting assay (Figure 4B). In addition, the β-catenin protein level decreased in a dose-dependent manner as well (Figure 4C). Infectious virus particles in the culture medium were quantified by virus titration using a TCID_50_ assay. Virus titers in 10μM iCRT14-treated cells were significantly reduced at 72 h p.i., compared to the control group (Figure 4D).

**Figure 5:**
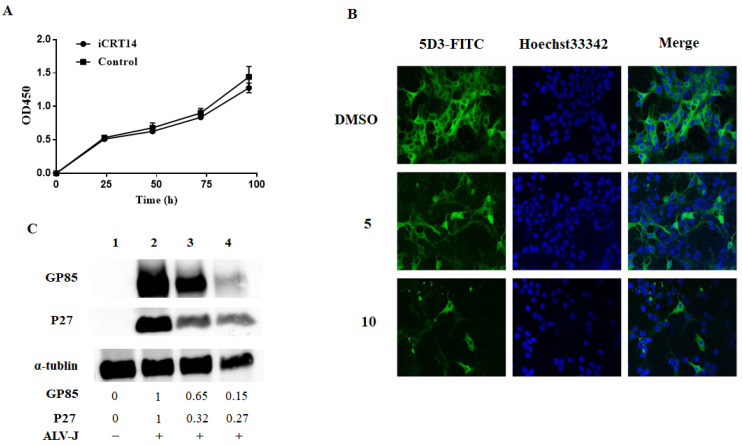
iCRT14 inhibited ALV-J replication in LMH cells. (**A**) LMH cells were treated with iCRT14 (10 μM) or vehicle control for various times (24, 48, 72, and 96 h), and the cell viability was detected by OD450 with CCK-8 kit. Data are expressed as the mean ± SD of three independent experiments. (**B**) LMH cells were infected with ALV-J in the presence of 5 or 10 μM of iCRT14 and DMSO. At 72 h p.i., the cells were fixed and stained for viral protein p27 (green) and the nuclei (blue). The pictures were captured and merged with a Leica SP2 confocal microscope (magnification, ×630). The results are representative of three independent experiments. (**C**) After 72 h p.i, the cell lysates were harvested for Western blotting. The control α-tubulin levels indicate that similar levels of protein were loaded into each lane, and the grayscale value of the bands in Western blot were analyzed with Image J software. Line 1: Nonvirus control group; line 2: DMSO group; line 3: 5 μM iCRT14 group; and line 4: 10 μM iCRT14 group. The immunoblotting experiments were performed independently at least three times with similar results.

In order to further confirm the effect of iCRT14, we also detected the ability of iCRT14 to inhibit virus replication in LMH cells by immunofluorescence and Western blotting assay. To rule out the possibility that the inhibition was due to cytotoxicity of iCRT14 on LMH cells, a CCK-8 cell viability assay was performed. The cell viability was detected by OD450 after treatment with iCRT14 at concentrations of 10 μM (Figure 5A). As expected, iCRT14 significantly reduced the viral protein expression in LMH cells (Figure 5B,C). Taken together, these results demonstrate that iCRT14 inhibits the Wnt/β-catenin signaling pathway and decreases ALV-J replication in CEF and LMH cells.

### 3.3. Knockdown of β-Catenin Inhibits ALV-J Replication

**Figure 6:**
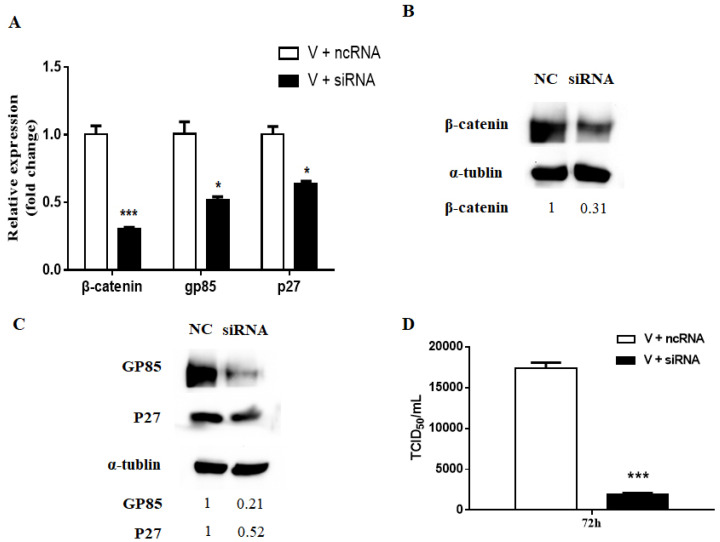
Knockdown β-catenin reduced ALV-J replication in CEF cells. The CEF cells were transfected with siRNA 986 targeting β-catenin (siRNA) or a control vector (ncRNA) for 24 h and then infected with ALV-J (MOI 1.0) for 72 h. (**A**) The gene expression levels of β-catenin, gp85, and p27 were tested with real-time PCR. The protein levels of β-catenin (**B**) and viral proteins, gp85 and p27, (**C**) were detected with Western blot. The control α-tubulin levels indicate that similar levels of protein were loaded into each lane, and the grayscale value of the bands in Western blot were analyzed with Image J software. (**D**) Virus titer were determined by TCID_50_ assay. Differences in gene expression and virus titer assay between the groups were assessed with an analysis of variance followed by Student’s *t*-tests. Data were expressed as mean ± SD from three independent experiments and analyzed (* *p* < 0.05, *** *p* < 0.001). These experiments were performed independently at least three times with similar results.

To further confirm the effects of the Wnt/β-catenin signaling pathway on ALV-J infection, we inhibited the pathway by knockdown of β-catenin using siRNA according to a previous report [19]. In our previous unpublished study, one siRNA 986 had interference capacity above 50% on β-catenin. Transfection of CEF cells with 986 siRNA resulted in a marked reduction of β-catenin gene and protein expression (Figure 6A,B). The inhibition of signaling pathway by knockdown of β-catenin significantly reduced gene and protein expression of gp85 and p27 (Figure 6A,C). Meanwhile, the virus proliferation was limited in CEF cells also (Figure 6D).

## 4. Discussion

The Wnt/β-catenin is a crucial signaling pathway in the growth of the body, cell differentiation, communication, and proliferation. It is associated and involved in many diseases, including cancer, neurological, autoimmune, and inflammation diseases [8,10]. Several reports have shown that different viruses regulate the Wnt/β-catenin signaling pathways through different mechanisms. Some viruses can activate or deactivate the Wnt/β-catenin pathway. These include viruses such as HIV [31], porcine circovirus-like virus P1 [21], HCMV [32], and HCV [17]. On the other hand, the Wnt/β-catenin signaling pathway has varying effects on different viruses. For example, the activation of the Wnt/β-catenin pathway by Wnt3a increased influenza virus replication in vitro and in vivo [18], and the stimulation of the Wnt/β-catenin pathway promoted the productive infection of bovine herpesvirus 1 [23]. Moreover, the Wnt/β-catenin signaling pathway inhibited PRRSV infection by regulating an NF-κB-dependent innate immune response [19] and activation of the Wnt/β-catenin signaling pathway by LiCl inhibits HIV replication and PRRSV infection [14,20].

In mammalian cells, Wnt ligands (Wnt3A and Wnt9B) are usually used to activate the Wnt/β-catenin signaling pathway and induce the expression of target genes. However, our unpublished studies showed that Wnt3A and Wnt9B with different concentrations had no significant effect on CEF cells. Based on the previous study about Wnt/β-catenin signaling pathway in chicken primordial germ cells [24], we tried to use GSK-3 inhibitor X, an acetoxime analog of BIO, to activate the signaling pathway. The results showed that GSK-3 inhibitor X could stabilize β-catenin and lead to target gene expression in CEF cells (Figure 1), which means GSK-3 inhibitor X did activate the signaling pathway. In the case of iCRT14, a small molecule inhibitor of the Wnt/β-catenin signaling pathway which displays minimal effect on the noncanonical Wnt/β-catenin signaling pathway and other pathways [30], it dramatically decreased β-catenin and target gene expression in CEF cells (Figure 3B). Here, we first evaluated and reported the effect of GSK-3 inhibitor X and iCRT14 on the Wnt/β-catenin signaling pathway in CEF cells. In the subsequent experiments, the GSK-3 inhibitor X increased ALV-J mRNA, protein expression, and virus production in CEF cells, while the iCRT14 inhibited virus replication significantly. These results are consistent with previous studies on how the Wnt/β-catenin pathway regulates influenza virus and bovine herpesvirus 1 [18,23]. In recent reports, inhibition of the Wnt/β-catenin pathway by downregulated β-catenin expression promoted viral replication [19,22]. However, in this study, ALV-J gp85 and p27 mRNA expression (Figure 6A), viral protein expression (Figure 6C), and virus titer (Figure 6D) were also significantly decreased in CEF cells transfected siRNA β-catenin compared with control siRNA transfected cells. These results suggested that the Wnt/β-catenin signaling pathway affects different viruses’ replication by different mechanisms. In addition, it is worth noting that in CEF cells, ALV-J infection obviously increased the total β-catenin protein level in cells without any treatment (Figure 4C), although this was not the focus of this study. This provides evidence for future research about how the virus regulates the Wnt/β-catenin signaling pathway.

In conclusion, this study provides the first evidence that the activation of the Wnt/β-catenin signaling pathway promotes ALV-J gene expression and virus production in CEF cells, and the inhibition of the Wnt/β-catenin signaling pathway limits virus production in both CEF and LMH cells. These findings provide new insights into the virus–host interaction of ALV-J and identified some potential antiviral strategies for ALV-J control.

## Figures and Tables

**Table 1:** Real-time PCR primer pairs.

Gene	Forward Primer (5′-3′)	Reverse Primer (5′-3′)
gp85	GTCTACAGTCAGCGACCTCA	GCTGGCTAAATCGGTGTTGT
p27	CCGGGGAATTGGTTGCTAT	AGTCAATGATCACCGGAGCC
β-catenin	ACAGCAAGGAACATGGCAAC	CCACTCAAAGAGGGAGCAGT
LEF1	CTTCAAGGACGAAGGGGACC	GTTGACCAGCGAGGACTTGA
TCF4	AAATCCCCCATCCGCTAGGA	AGCCGACGTCACTCTGGGAA
Axin	GCTTCAGGCAGATGGACCTT	CTTGAGCTGCTTGGAGACGA
APC	TGCTTGCGGAACTTGAGAAG	GCCTCATATTCCAGCTGCCT
CyclinD1	GCACAGCAGCACAACGTATC	ATCTCGCACATCAGTGGGTG
c-myc	CAGAGGAGCACTGTAAGCCC	AGCAGCGTAGTTGTGTTGGT
18S	TCAGATACCGTCGTAGTTCC	TTCCGTCAATTCCTTTAAGTT

## Data Availability

All datasets generated for this study are included in the article.
